# Hashimoto’s thyroiditis in differentiated thyroid cancer: autoimmune inflammation, immune surveillance, and tumor escape

**DOI:** 10.3389/fimmu.2026.1945791

**Published:** 2026-10-16

**Authors:** Zijian Zhang, Likun Cui, Quan Dai, Chuanshi He, Li Han, Min Zhuang, Lu Wang, Ziyue Hu, Xiaoxiao Xie, Yuping Shen, Jinshun Xu, Man Lu

**Affiliations:** 1 Department of Ultrasound, Sichuan Clinical Research Center for Cancer, Sichuan Cancer Hospital & Institute, Sichuan Cancer Center, School of Medicine, University of Electronic Science and Technology of China, Chengdu, China; 2 Department of Ultrasound, Sichuan Clinical Research Center for Cancer, Sichuan Cancer Hospital & Institute, Sichuan Cancer Center, University of Electronic Science and Technology of China, Chengdu, China

**Keywords:** differentiated thyroid cancer, Hashimoto’s thyroiditis, immune surveillance, papillary thyroid carcinoma, redifferentiation therapy

## Abstract

Hashimoto’s thyroiditis (HT) and differentiated thyroid cancer (DTC) frequently coexist, yet their biological relationship remains paradoxical. In this critical narrative review informed by a structured literature search, we synthesize epidemiological, clinicopathological, genomic, immunological, and translational evidence derived predominantly from papillary thyroid carcinoma (PTC) within the broader DTC framework to examine how HT may function as both a tumor-promoting inflammatory niche and a tumor-restraining immune microenvironment. In the proposed spear-and-shield model, chronic autoimmune inflammation may generate oxidative stress, cytokine signaling, and selective pressures that favor malignant transformation, whereas pre-existing lymphocytic infiltration, antigen presentation, B-cell organization, and cytotoxic T-cell activity may strengthen immune surveillance and contribute to favorable outcomes in selected patients. We further discuss how immune escape can emerge within an inflamed thyroid microenvironment through checkpoint activation, regulatory lymphocytes, myeloid polarization, dendritic-cell remodeling, and thyroid-stimulating hormone/thyroid-stimulating hormone receptor (TSH/TSHR)-related immune modulation. Finally, because most mechanistic and clinical evidence derives from papillary thyroid carcinoma, we emphasize that HT should be characterized according to its timing, histological features, antibody profile, thyroid functional status, and spatial immune organization. We propose that HT influences DTC through a stage- and genotype-dependent immune contexture rather than as a uniform risk factor, and identify testable biomarkers integrating T-cell and B-cell receptor clonality, tertiary lymphoid structure maturity, antigen presentation, checkpoint activation, thyroid differentiation, and TSH–TSHR signaling.

## Introduction

The incidence of differentiated thyroid cancer (DTC), particularly papillary thyroid carcinoma (PTC), has increased substantially worldwide over recent decades (1). Although enhanced surveillance and widespread ultrasonography have contributed to the detection of indolent microcarcinomas (1, 2), evidence also suggests a genuine increase in disease burden associated with environmental and lifestyle factors (3). Despite the generally favorable prognosis of DTC, a clinically important subset of patients develops recurrent, metastatic, or radioiodine-refractory disease.

In parallel, Hashimoto’s thyroiditis (HT) is the most prevalent organ-specific autoimmune disorder (4, 5). It is characterized by dense lymphocytic infiltration, predominantly involving CD4^+^ T cells, CD8^+^ T cells, and B cells, together with the production of anti-thyroid peroxidase (TPOAb) and anti-thyroglobulin (TgAb) antibodies (6). Thus, HT represents a chronic inflammatory state of the thyroid rather than a passive clinical comorbidity. The frequent clinical convergence of HT and DTC, with reported coexistence rates varying widely across surgical and histopathological cohorts (7), has sparked intense debate regarding their biological interrelationship.

This coexistence raises a central “spear-and-shield” paradox: chronic inflammatory signals in HT may act as a “spear” that promotes tumor initiation, whereas pre-existing autoimmune responses may serve as a “shield” that enhances immune surveillance and restrains tumor progression. In this critical narrative review informed by a structured literature search, we integrate epidemiological, clinicopathological, genomic, immunological, and translational evidence to develop a stage- and genotype-dependent conceptual framework for interpreting the relationship between HT and DTC. Through this framework (**Figure 1**), we aim to support immune-informed risk stratification, improve diagnostic interpretation, and highlight therapeutic strategies that selectively harness beneficial autoimmunity while limiting chronic inflammation. Thus, the key issue is not whether HT is inherently protective or harmful, but how the phenotype and timing of thyroiditis interact with antigen specificity, immune-cell composition, spatial immune organization, tumor genotype, histological subtype, thyroid functional status, and differentiation state to determine whether autoimmune inflammation promotes tumor evolution or supports effective immune surveillance. In this review, HT is therefore not treated as a binary comorbidity. We distinguish serological autoimmunity, histologically confirmed lymphocytic thyroiditis, and ultrasound-defined thyroiditis; analyze adult and pediatric disease separately; and interpret clinical associations in the context of surveillance bias, tumor genotype, histological subtype, and differentiation state. This distinction is essential because the immune infiltrate that detects or restrains an early thyroid tumor may differ fundamentally from the inflammatory infiltrate remodeled by an established, aggressive, or dedifferentiated tumor. PTC is the predominant histological subtype of DTC and provides the most extensive clinical and mechanistic evidence concerning concurrent HT. Accordingly, PTC serves as the principal analytical anchor of this review, whereas DTC is retained as the overarching clinical framework. Evidence from follicular thyroid carcinoma and oncocytic thyroid carcinoma is discussed where available. For clarity, PDTC, differentiated high-grade thyroid carcinoma, and ATC are treated as distinct high-grade or undifferentiated entities and are not classified as DTC in this review.

**Figure 1:**
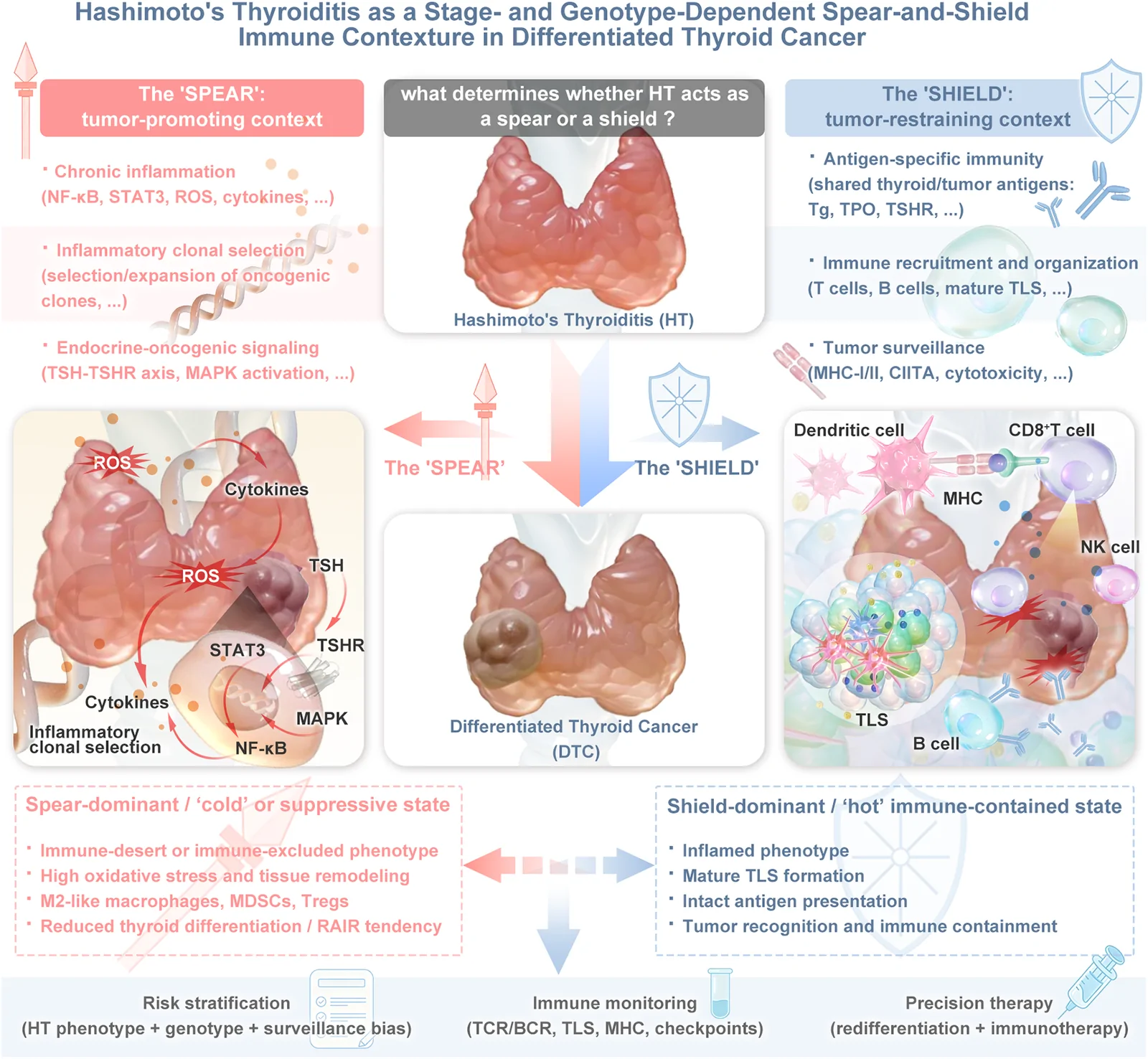
Hashimoto’s thyroiditis as a stage- and genotype-dependent spear-and-shield immune contexture in differentiated thyroid cancer. Hashimoto’s thyroiditis (HT) may shape differentiated thyroid cancer (DTC) through opposing but interconnected immune and inflammatory programs. The “spear” component reflects chronic autoimmune inflammation, reactive oxygen species, cytokine signaling, tissue remodeling, compensatory thyrocyte proliferation, inflammatory clonal selection, expansion of oncogenic clones, immune escape, and reduced thyroid differentiation. These processes may favor clonal expansion, immune evasion, radioiodine-refractory tendency, and tumor progression. The “shield” component reflects antigen-specific immunity, intact MHC-I/MHC-II antigen presentation, cytotoxic effector function, B-cell and tertiary lymphoid structure organization, and resistance to checkpoint-mediated exhaustion. These processes may support tumor recognition, immune containment, and an indolent phenotype. Whether HT acts predominantly as a spear or a shield depends on HT timing, diagnostic phenotype, tumor subtype, tumor genotype, spatial immune organization, differentiation state, and treatment context. Because most evidence derives from papillary thyroid carcinoma, HT should not be interpreted as a uniform risk factor but as a dynamic immune contexture requiring biomarker integration across TCR/BCR clonality, TLS maturity, antigen presentation, checkpoint activation, thyroid differentiation, and TSH–TSHR signaling.

These advanced entities are included selectively as thyroid-lineage contextual evidence because they illustrate how high-grade progression and dedifferentiation may alter antigen presentation, immune-cell composition, checkpoint dependence, thyroid-lineage antigen expression, and therapeutic responsiveness. A subset of ATCs may arise through genomic and transcriptomic progression from pre-existing PTC; however, we do not imply that all PDTCs or ATCs follow a uniform linear evolution from PTC. Findings from these advanced subtypes are therefore interpreted as indirect mechanistic or translational evidence rather than as direct evidence from HT-associated PTC.

To clarify the specific contribution of this review, **Table 1** summarizes how our approach extends previous reviews on HT and thyroid cancer. In contrast to previous reviews focused mainly on HT–PTC coexistence, clinicopathological associations, prognosis, or general immunotherapeutic opportunities, the present review integrates HT phenotype and timing, surveillance bias, tumor genotype, immune organization, and differentiation state within a stage- and genotype-dependent framework.

**Table 1:** Distinctive scope and added value of this review compared with previous reviews.

Dimension	Common focus in previous reviews	Added perspective in this review
Overall framework	HT–PTC coexistence, risk, prognosis, or “double-edged sword” effects.	A stage- and genotype-dependent spear-and-shield model of HT-associated DTC.
HT definition and timing	HT often treated as a present/absent comorbidity.	Distinguishes serological, histological, and ultrasound-defined thyroiditis, with attention to timing and surveillance bias.
Cancer subtype and genotype	Strong emphasis on PTC and BRAF-related associations.	Clarifies the PTC-dominant evidence base and integrates tumor subtype, fusions, TERT alterations, and differentiation state.
Immune mechanism	Lymphocytic infiltration, PD-L1 expression, or general immune microenvironment.	Emphasizes immune quality, spatial organization, antigen specificity, TCR/BCR clonality, B-cell/TLS maturity, and exhaustion.
Comparative autoimmunity	Spontaneous HT and ICI-induced thyroiditis usually discussed separately.	Uses ICI-induced thyroiditis as a comparative model, while avoiding direct equivalence with spontaneous HT.
Translational integration	Immunotherapy or redifferentiation therapy often reviewed separately.	Links MHC-I/MHC-II/CIITA antigen presentation, redifferentiation, thyroid target expression, and precision immunotherapy.

BCR, B-cell receptor; CIITA, class II transactivator; DTC, differentiated thyroid cancer; HT, Hashimoto**’**s thyroiditis; ICI, immune checkpoint inhibitor; MHC, major histocompatibility complex; PTC, papillary thyroid carcinoma; TCR, T-cell receptor; TLS, tertiary lymphoid structure.

## Review methodology

This review was conducted as a critical narrative review informed by a structured literature search. A narrative design was selected because the objective was to integrate heterogeneous evidence spanning epidemiology, clinicopathology, tumor genomics, basic and translational immunology, single-cell and spatial profiling, endocrine signaling, and therapeutic studies. The review was therefore intended to develop a stage- and genotype-dependent conceptual framework for the relationship between Hashimoto**’**s thyroiditis (HT) and differentiated thyroid cancer (DTC), rather than to address a narrowly predefined intervention question, generate pooled effect estimates, or provide an exhaustive systematic mapping of a methodologically uniform evidence base.

To support the narrative synthesis, PubMed/MEDLINE, Web of Science Core Collection, and Scopus were searched from database inception to 22 July 2026. No language restriction was applied during database searching; however, only English-language full-text publications were included during eligibility assessment. The primary search combined terms related to autoimmune thyroiditis, including **“**Hashimoto thyroiditis,**” “**Hashimoto disease,**” “**chronic lymphocytic thyroiditis,**” “**autoimmune thyroiditis,**”** and **“**thyroid autoimmunity,**”** with terms related to follicular-cell-derived thyroid cancer, including **“**thyroid cancer,**” “**thyroid carcinoma,**” “**differentiated thyroid cancer,**” “**papillary thyroid carcinoma,**” “**follicular thyroid carcinoma,**”** and **“**oncocytic thyroid carcinoma.**”** Additional topic-specific searches incorporated terms related to epidemiology and surveillance bias; prognosis and recurrence; BRAF, RAS, RET/PTC, oncogenic fusions, TERT, and MAPK signaling; the tumor immune microenvironment; T-cell, B-cell, macrophage, dendritic-cell, and natural-killer-cell responses; antigen presentation, MHC-I/MHC-II, TCR/BCR clonality, and tertiary lymphoid structures; single-cell and spatial profiling; PD-1/PD-L1 signaling and immune checkpoint inhibitor-induced thyroiditis; TSH–TSHR signaling; thyroid differentiation and radioiodine refractoriness; redifferentiation therapy; and immunotherapy. The reference lists of relevant reviews and eligible primary studies were manually screened, and forward citation tracking was used to identify additional influential or recently published studies. The complete database-specific search strategy is provided in **Supplementary Table S1**. For thematic synthesis, evidence was classified into three categories. Direct evidence included clinical or experimental studies specifically examining HT-associated PTC or, where available, broader cohorts of HT-associated DTC. Thyroid-lineage contextual evidence included studies of PTC or DTC without HT stratification and studies involving PDTC, differentiated high-grade thyroid carcinoma, ATC, or other advanced thyroid malignancies. These studies were used to interpret the biological consequences of high-grade progression, dedifferentiation, immune remodeling, antigen-presentation loss, and therapeutic target preservation. Evidence derived from non-thyroid malignancies was considered hypothesis-generating. Conclusions concerning HT-associated thyroid cancer were based primarily on direct evidence, whereas contextual and cross-cancer evidence was explicitly qualified and was not used to infer clinical efficacy in HT-associated DTC.

Eligible publications included peer-reviewed clinical cohort, case-control, cross-sectional, and prospective studies; experimental studies using cellular or animal models; genomic, transcriptomic, proteomic, single-cell, spatial, and other multi-omics studies; systematic reviews and meta-analyses; major clinical guidelines; and highly relevant translational studies. Studies directly examining HT or autoimmune thyroiditis in relation to DTC or papillary thyroid carcinoma were prioritized. Studies involving poorly differentiated thyroid carcinoma, anaplastic thyroid carcinoma, or non-thyroid malignancies were included selectively only when they addressed mechanisms directly relevant to DTC progression, dedifferentiation, antigen presentation, immune escape, endocrine–immune signaling, or therapeutic response. Evidence from these indirect settings was explicitly interpreted as contextual or hypothesis-generating rather than as equivalent to direct evidence from HT-associated DTC. Studies focused primarily on medullary thyroid carcinoma, benign thyroid disorders unrelated to autoimmunity, conference abstracts, non-peer-reviewed reports, duplicate publications, articles without accessible full text, and publications lacking direct clinical or mechanistic relevance were excluded.

The primary database search and title/abstract screening were performed by Zijian Zhang. Potentially relevant full-text articles and the final set of included publications were independently reviewed by Likun Cui. Any disagreements regarding eligibility or interpretation were resolved through discussion and consensus, with consultation of the corresponding authors, Jinshun Xu and Man Lu, when necessary. Clinical associations were interpreted with greater weight given to systematic reviews, meta-analyses, prospective studies, and large cohort studies, whereas mechanistic conclusions were prioritized when supported by functional experimental models or high-resolution molecular evidence. Because of substantial heterogeneity in HT definitions, study populations, tumor subtypes, experimental designs, and clinical endpoints, no quantitative meta-analysis, formal risk-of-bias assessment, or certainty-of-evidence grading was performed.

## Epidemiological and clinical paradox of HT-DTC coexistence

### Histological spectrum and clinical risk in DTC

The global incidence of thyroid cancer (TC) has steadily increased over recent decades, largely driven by DTC (4, 8–10). In this review, DTC refers to conventional differentiated follicular-cell-derived carcinomas, principally PTC, follicular thyroid carcinoma (FTC), and oncocytic thyroid carcinoma. Differentiated high-grade thyroid carcinoma (DHGTC), PDTC, and ATC are considered distinct high-grade or undifferentiated entities rather than components of the direct HT-associated DTC evidence base. They are discussed selectively to provide biological context for lineage progression, loss of thyroid differentiation, immune remodeling, and treatment resistance (3, 11, 12). Widespread neck ultrasonography has increased the detection of thyroid nodules, which are subsequently evaluated by cytopathologic analysis for early diagnosis. Although PTC is generally indolent, a subset of patients develops aggressive, recurrent, or radioiodine-refractory disease. Therefore, accurate risk stratification remains essential to reduce both overdiagnosis and undertreatment (4, 13).

Most PTCs and FTCs have favorable outcomes, with a 5-year progression-free survival (PFS) exceeding 95%. However, high-risk DTC accounts for approximately 5-10% of cases (14, 15). Distinct from well-differentiated DTC, PDTC and DHGTC occupy an intermediate biological spectrum between DTC and anaplastic thyroid cancer (ATC). Some tumors have also been reclassified as DHGTC according to the 2022 WHO criteria (16). These entities are associated with substantially poorer prognosis, with 5-year and 10-year overall survival (OS) generally limited to 50% to 80% (12, 15). Established adverse prognostic factors include larger tumor size, older age, extrathyroidal invasion, lymph node metastasis, and distant metastasis.

### PTC-centered clinical evidence in HT-associated DTC: prognostic divergence and surveillance bias

HT is the most prevalent autoimmune thyroid disease and is clinically characterized by elevated serum anti-thyroid peroxidase antibody (TPOAb), anti-thyroglobulin antibody (TgAb), and intrathyroidal immune infiltration resulting from failed immune tolerance (6, 17–19). Thyroid hormone replacement is the standard treatment for clinical and subclinical hypothyroidism. The prevalence of HT varies by sex, age, geographic region, and iodine supplementation (19). HT is also frequently associated with systemic autoimmune diseases, including systemic lupus erythematosus (SLE), Sjögren’s syndrome (SS), systemic sclerosis (SSc), and rheumatoid arthritis (RA). Separately, thyroiditis and thyroid dysfunction may occur as immune-related adverse events during cancer immunotherapy, although these entities are mechanistically distinct from spontaneous HT (20).

The co-occurrence of HT and DTC has been extensively studied but remains debated. Among patients with PTC, the proportion with concurrent HT is higher than that in the general population, ranging from 6% to nearly 80% across studies (7). This wide variation may reflect differences in geography, iodine intake, histological subtype, patient selection, diagnostic criteria, and tumor clinicopathological features. Numerous studies have reported that PTCs coexisting with HT tend to be smaller and often present as microcarcinomas (7, 21). The relationship between HT and major DTC histological subtypes also warrants attention. Yang et al. (22) and Liu et al. (23) et al. reported a significant association between HT and thyroid cancer histology, with PTC being the dominant subtype among patients with concurrent HT. In pediatric populations, high-risk PTC variants appear more frequent in patients with HT (24, 25), and children or adolescents with HT may more often present with familial PTC (26).

A national cohort study from Korea reported that autoimmune diseases, particularly thyroid-specific autoimmune diseases such as HT and Graves’ disease (GD), were associated with an increased risk of thyroid malignancy (27). Similarly, a systematic review incorporating 11 case-control studies and 12 cohort studies reported an increased risk of thyroid cancer among patients with HT (28). However, this association should be interpreted cautiously, because intensified surveillance and frequent ultrasonography in patients with HT may increase the detection of indolent thyroid microcarcinomas (29). Thus, the observed association between HT and thyroid cancer may reflect both biological susceptibility related to immune dysregulation and surveillance-related overdiagnosis.

The prognostic impact of HT in DTC remains inconsistent across studies. In children and young adults, coexisting HT has been associated with regional metastasis and recurrence (30, 31). Other studies reported a higher frequency of central compartment lymph node metastasis in HT-DTC, whereas lateral neck lymph node metastasis was not consistently significant (7, 17, 32). In contrast, several studies found that patients with coexisting HT presented with less aggressive characteristics and better outcomes (7, 17, 21, 33–36). Supporting this favorable view, De Leo et al. (38) showed that coexisting HT does not increase the risk of structural disease recurrence. A meta-analysis including 28,143 patients showed that HT was associated with favorable prognostic features in PTC regardless of BRAF status (17). This disconnect between initial tumor characteristics and long-term outcomes has led to the proposal that HT acts as a “double-edged sword,” potentially promoting thyrocyte proliferation while restraining lymph node invasion in selected contexts (35).

Treatment-related implications should be distinguished from prognostic associations. Current evidence is insufficient to support modifying surgery, radioactive iodine therapy, or TSH-suppression strategies solely because HT is present. The clearest clinical impact concerns postoperative response assessment, as TgAb may interfere with thyroglobulin surveillance and may produce biochemical abnormalities without corresponding structural recurrence (25, 38). Autoimmune thyroiditis may also influence radioiodine uptake and inflammatory responses, although evidence for differential therapeutic benefit remains limited (119). Prognostically, cohort studies and meta-analyses generally suggest neutral or favorable outcomes in HT-associated PTC, although the findings vary according to age, tumor genotype, HT definition, and surveillance intensity (17, 21, 33–38). A published TCGA-THCA analysis of 343 PTC cases with a recorded history of thyroiditis also reported a lower recurrence probability (OR = 0.24, 95% CI = 0.03–1.00, P = 0.038) (103). However, this annotation does not represent standardized HT phenotyping, and the result should not be extrapolated to all DTC subtypes.

To overcome these diagnostic and prognostic ambiguities, radiomics and AI-based models may further refine risk interpretation. Multiregional ultrasonomic signatures have shown favorable calibration for predicting malignancy in thyroid nodules with coexisting HT (7), and Chen et al. (37) developed an HT nodule-AI model with improved diagnostic performance, offering a promising tool to navigate the complex ultrasound landscape altered by chronic inflammation. Whether ultrasound-based signatures can reflect CD8^+^ T-cell infiltration or tertiary lymphoid structure maturity in HT-associated PTC remains to be validated. Thus, the epidemiological literature should be read as a paradox rather than a contradiction: HT may increase cancer detection and, in some settings, reflect early tumor-promoting inflammation, while simultaneously marking an immune-active state that constrains progression in selected patients.

## The spear: autoimmune inflammation as a tumor-promoting niche

### Genomic susceptibility and inflammation-driven clonal selection

A potential tumor-promoting role of HT can be conceptualized as an inflammatory selection process rather than as a direct carcinogenic event. Chronic thyroiditis may generate oxidative stress, cytokine and chemokine signaling, tissue remodeling, and compensatory thyrocyte proliferation, thereby creating conditions that could favor the survival or expansion of oncogenic clones. However, direct longitudinal evidence that spontaneous HT drives clonal evolution in humans remains limited, and the expansion of such clones would likely depend on their genotype, antigenicity, differentiation state, and capacity to evade immune elimination. This selection-based view helps reconcile why HT may be associated with increased PTC detection in some cohorts yet with less aggressive clinicopathological features in others. Although thyroid follicular cells generally have a low mutational burden, the major initiating drivers of DTC, including BRAFV600E, RAS mutations, and selected receptor tyrosine kinase fusions or rearrangements, converge largely on mitogen-activated protein kinase (MAPK) signaling. By contrast, TERT promoter mutations and other late progression-associated alterations are better interpreted as modifiers of dedifferentiation, metastatic competence, radioiodine-refractory progression, and adverse prognosis rather than as primary MAPK-activating events. Together, these tumor-intrinsic programs shape histopathology, differentiation state, metastatic behavior, and treatment responsiveness (12, 14, 40–42). Clinically, these pathway alterations are associated with regional lymph node (LN) metastasis, tumor progression, and a higher proportion of radioiodine-refractory (RAIR) disease (4, 9, 14, 39).

At the transcriptomic level, PDTC often exhibits a RAS-like profile (40). In contrast, PTCs with high-grade features show a higher frequency of BRAFV600E mutations than PDTCs, a profile that correlates with more advanced TNM stage and poorer disease-specific survival (DSS) (43). Interestingly, the prognostic impact of the immune microenvironment appears closely linked to this genetic background. While aggressive genetic profiles drive disease progression, coexisting HT has been associated with less aggressive tumors and has been reported as an independent protective factor against recurrence, particularly in BRAF-wild type DTC (44).

A large-scale review integrating data from 47 studies reported that patients with HT were nearly 50% less likely to harbor BRAF-mutated thyroid cancer than patients without HT (17). Consistently, PTCs co-occurring with HT have been associated with fewer BRAF V600E mutations and more favorable prognostic profiles (17, 52). Within PTC with concurrent HT, BRAF-mutated tumors were associated with more frequent lymph node metastasis and extrathyroidal extension than BRAF-wild-type tumors (53). Conversely, among patients with BRAF-wild-type DTC, HT was reported as a protective factor against tumor aggressiveness and recurrence (44). These findings suggest that HT may modify PTC progression in a genotype-dependent manner, rather than acting as a uniformly protective factor (17, 53).

At the molecular level, the genetic architecture of HT itself is shaped by inherited susceptibility, environmental factors, and epigenetic regulation (6, 45, 46). Compared with earlier case-control studies, genome-wide association studies (GWAS) have identified multiple autoimmune thyroid disease (AITD) susceptibility loci, including IL2RA, HLA, PTPN22, TSHR, TPO, FOXE1, and CTLA4 (47, 48). These loci implicate viral triggers, aberrant T-cell and B-cell signaling, and thyroid-specific immune pathways in thyroiditis. When HT coexists with PTC, it may also be associated with changes in the tumor molecular landscape. For instance, MET and FAM20A have been identified as hub genes with potential diagnostic value in PTC with coexisting HT, highlighting alterations in epithelial-mesenchymal transition (EMT) and PI3K-Akt signaling (49, 50). Additionally, proteomic profiling has identified 44 differentially expressed proteins in HT, primarily involving cell adhesion and energy metabolism (51).

The interaction between genetic profiles and autoimmunity is also highly dependent on demographic context. While HT often correlates with an indolent tumor profile in adults, this protective paradigm does not universally apply. In a recent Chinese pediatric PTC cohort, RTK fusions were detected in 25% of cases and were significantly associated with coexisting HT, diffuse sclerosing subtype, and extrathyroidal extension, contrasting with the indolent profile typically observed in adults (23). In this cohort, neither BRAF mutation nor autoimmune status significantly affected recurrence, highlighting the clinical heterogeneity of HT-associated DTC.

### Oncogenic fusions and immune-inflammatory signaling hubs

In a cohort of RET fusion-positive thyroid cancers, concomitant HT was more frequent in the CCDC6-RET group than in the NCOA4-RET group (67.9% vs. 22.7%) (54). Experimental studies further support a mechanistic link between oncogenic fusions, thyroid autoimmunity, and tumor selection. For instance, Denning et al. (19, 55) showed that RET/PTC1 expression alters the immunophenotype of thyroid follicular cells, potentially allowing transformed cells to survive within the immune-inflammatory environment of HT. Consistently, RET/PTC1 expression in normal human thyroid cells induces inflammatory and invasion-related programs, including the upregulation of cytokines, chemokines, matrix-degrading enzymes, and adhesion molecules (56, 57). RET/PTC3 has also been implicated in both thyroid tumorigenesis and autoimmune thyroiditis, suggesting that RET/PTC rearrangements may serve as molecular bridges between oncogenic transformation and inflammation (57).

Beyond specific gene fusions, shared inflammatory signaling hubs and microenvironmental interactions further connect HT and DTC. NF-κB emerges as a central regulator of thyroid biology, autoimmunity, and cancer (58–60). In thyroiditis, NF-κB activation facilitates antigen presentation by thyrocytes and amplifies innate and adaptive immune responses; in cancer, however, aberrant NF-κB signaling promotes cellular proliferation, apoptosis resistance, and inflammation-driven tumor progression. RTK fusions in pediatric DTC, particularly when associated with HT and aggressive features such as diffuse sclerosing subtype and extrathyroidal extension, further support the “spear” paradigm in selected populations (23). Recent advances in single-cell RNA sequencing have provided higher-resolution insight into this microenvironment, identifying HT-associated cell populations (HASCs) in PTC. These populations may modulate tumor behavior through TSH-related effects and complex immune-stromal interactions, notably involving the MIF-CD74/CXCR4 axis (61).

Several shared molecular candidates have also been identified through transcriptomic and bioinformatic analyses. Liu et al. (62) identified four hub genes—CLTA, EDIL3, HAPLN1, and HIP1—implicated in both thyroid cancer and HT. Similarly, Zhang et al. (63) reported significant genetic overlap, highlighting shared somatic mutations and immune-related genes, such as BACH2 and RNASET2, between HT and PTC. Additional studies have identified LTF, CCL21, SERPINA1, DMBT1, and AHNAK2 as key mediators linking HT and PTC; notably, SERPINA1 and DMBT1 correlate with favorable prognoses, whereas AHNAK2 is linked to advanced disease, poor survival, and immune-cell infiltration (64–67). Collectively, these findings support an immune selection model: autoimmune inflammation may favor the emergence of specific oncogenic clones equipped to exploit inflammatory pathways, while the same immune pressure may eliminate or restrain clones that remain sufficiently immunogenic.

## The shield: immune surveillance and beneficial autoimmunity

### Immune surveillance in the HT-associated tumor microenvironment

Multiple immune and stromal populations within the thyroid cancer microenvironment shape tumor progression, prognosis, and immune evasion (68). Their biological effects depend on their functional states, spatial distribution, and interactions with tumor cells rather than on cellular abundance alone (18, 61, 69–78). In non-medullary thyroid cancer, immune dysregulation may begin before overt tumor infiltration, with altered myeloid-cell and bone marrow progenitor states, tolerant monocyte phenotypes, increased reactive oxygen species (ROS), and skewing toward lymphopoiesis (79).

In HT-associated PTC, however, this immune background may also support tumor restraint. PTCs with concomitant HT are often smaller, which may reflect either enhanced surveillance or immune-mediated tumor control (7). PTCs with concomitant HT have been associated with an inflamed tumor immune microenvironment characterized by increased CD8^+^ T-cell infiltration and STAT6 expression compared with PTC without HT (80). These findings support an association between HT and a distinct immune phenotype, but do not establish an HT-driven STAT6/CD8^+^ mechanism (80). Enhanced anti-tumor immunity within lymph node metastases, including increased Granzyme-B^+^ cells and decreased COX2, may also help explain why nodal involvement is not always an independent predictor of poor prognosis (82).

Nevertheless, the HT-associated immune microenvironment is not uniformly protective. IDO expression in papillary thyroid microcarcinoma correlates with FOXP3^+^ regulatory T cells and immune disruption, suggesting a role in tumor progression (83). NK-cell activity is often reduced in autoimmune thyroid diseases, and NK-cell cytotoxicity in thyroid cancer can be suppressed by factors such as TGF-β1 (77). Unlike Graves’ disease, in which activated NK cells may promote a protective M1 macrophage phenotype against DTC, HT has been associated with reduced NK activity and a more pro-tumor M2 macrophage environment (84). These observations suggest that shifting macrophage polarization from M2-like suppressive states toward M1-like cytotoxic states may represent a potential immunotherapeutic strategy.

Single-cell and immune-profiling studies further highlight the heterogeneity of HT-associated DTC. Risk models incorporating DCs, Tregs, γδ T cells, DC apoptosis, TCR-related pathways, HLA expression, and PD-L1 have been used to stratify recurrence risk in DTC (85). Progressive PTC may generate an immunosuppressive niche driven by LAMP3^+^ DCs, which promote CD8^+^ T-cell exhaustion and Treg infiltration (86). TIL subtypes also differ among PTC, HT, and normal tissues (87, 88), with distinct immunological subtypes of PTC and ATC defined by macrophage differentiation and metabolic-immune features (89, 90). Single-cell analysis further suggests that HT can create a TSH-suppressive niche within the microenvironment, while organoid studies indicate that thyroid follicular cells in HT actively promote immune-cell infiltration through CCL2 and CCL3 upregulation and activation of NF-κB and IFN-γ-CXCL10 signaling (19, 51, 61, 91).

Taken together, these findings shift the central question from whether HT is protective to which immune states within HT are protective, suppressive, or permissive for tumor progression. Therefore, the protective component of HT is unlikely to be determined by lymphocyte abundance alone, but rather by the quality, spatial accessibility, and antigen specificity of the immune response, including intact antigen presentation, cytotoxic effector function, organized B-cell/TLS niches, and resistance to checkpoint-mediated exhaustion. **Table 2** summarizes the major immune components and representative evidence shaping this spear-and-shield balance in HT-associated DTC.

**Table 2:** Immune components and representative evidence shaping the spear-and-shield balance in HT-associated DTC.

Immune component/pathway	Role in the spear-and-shield balance	Representative evidence	Clinical/translational implication
CD8^+^ T cells and cytotoxic lymphocytes	Pre-existing cytotoxic infiltration may restrain tumor expansion, whereas exhausted or spatially excluded T cells may permit immune escape.	(80, 82, 86, 105)	Assess cytotoxic activity, exhaustion markers, and spatial localization rather than lymphocyte density alone.
B cells and tertiary lymphoid structures (TLS)	B-cell infiltration and TLS may support antigen presentation and durable surveillance, but chronic B-cell activation may also sustain inflammation.	(74, 105, 176)	TLS organization and B-cell migration may refine prognosis in HT-associated DTC.
Macrophages, MDSCs, and myeloid programs	M2-like macrophages and MDSCs may promote angiogenesis, invasion, and immune suppression; M1-like polarization may support tumor clearance.	(79, 84, 89, 90, 148)	Myeloid polarization should be integrated with T-cell-based immune scores and therapeutic stratification.
Dendritic cells and antigen presentation	Efficient antigen presentation may expose shared thyroid/tumor antigens, whereas LAMP3^+^ or tolerogenic DC states may drive exhaustion and Treg recruitment.	(49, 86, 95, 106)	DC subsets may identify immune-active but suppressive tumors and guide checkpoint or vaccine strategies.
PD-1/PD-L1 and checkpoint signaling	Checkpoint activation dampens anti-tumor T-cell function and facilitates immune escape; checkpoint disruption can reveal thyroid- and tumor-directed immunity.	(92, 97–99, 123)	Checkpoint markers require interpretation within HT status, inflammatory context, and thyroiditis risk.
TSH-TSHR signaling	Promotes proliferation and PD-L1, but the T-cell exhaustion link is unproven; HT immunity may decouple TSH from adverse outcomes.	(61, 180, 187, 189)	Integrate TSH/TSHR, autoantibodies, and immune profiles, avoiding claims of TSH-driven exhaustion lacking thyroid-specific evidence.

DC, dendritic cell; DTC, differentiated thyroid cancer; HT, Hashimoto’s thyroiditis; MDSC, myeloid-derived suppressor cell; MHC, major histocompatibility complex; PD-1, programmed cell death protein 1; PD-L1, programmed death-ligand 1; TCR, T-cell receptor; TgAb, anti-thyroglobulin antibody; TLS, tertiary lymphoid structure; TPOAb, anti-thyroid peroxidase antibody; Treg, regulatory T cell; TSH, thyroid-stimulating hormone; TSHR, thyroid-stimulating hormone receptor.

## Immune escape within an inflamed thyroid microenvironment

### PD-1/PD-L1 signaling: from immune tolerance to tumor evasion

PD-1/PD-L1 signaling provides a mechanistic link between immune tolerance, thyroid autoimmunity, and tumor immune escape. In autoimmune thyroid disease, insufficient checkpoint-mediated tolerance may facilitate thyroid-directed immune injury, whereas in thyroid cancer, tumor exploitation of the same pathway can suppress anti-tumor immunity and promote immune evasion (18, 68, 92). Activation of PD-1/PD-L1 signaling in the tumor microenvironment suppresses T-cell activity, promotes regulatory T-cell differentiation, and enhances cancer-cell resistance to cytotoxic T lymphocyte (CTL)-mediated killing. Thus, the thyroid represents a useful model for studying how immune tolerance can be disrupted in autoimmunity yet co-opted during tumor progression.

Immune checkpoint inhibitor (ICI)-induced thyroiditis provides a mechanistically distinct example of checkpoint-disinhibited thyroid injury rather than direct evidence for the antitumor effects of spontaneous HT; its differences from spontaneous HT and HT-associated DTC are summarized separately in **Table 3** (46, 81, 93–96, 101, 102**).** In PTC, high PD-1/PD-L1 expression despite CTL infiltration suggests that an inflamed tumor microenvironment can remain functionally suppressed. PD-1/PD-L1 upregulation facilitates immune evasion by inactivating CTLs and is associated with lymph node metastasis (97, 98). Santana et al. (99) reported that PD-L1 expression was not determined by conventional tumor characteristics but was independently predicted by the inflammatory microenvironment, particularly HT with oncocytic metaplasia. Furthermore, PD-L1 expression has also been reported in HT-associated follicular epithelial dysplasia, a lesion proposed as a potential PTC precursor (98). These cross-sectional observations do not establish a temporal transition from HT to PTC (18, 98). Elevated PD-1 expression on peripheral T cells may also help distinguish PTC from benign goiter, with the highest levels observed in patients with concurrent HT (100). Although HT-associated PTC and immunotherapy-induced thyroid dysfunction may share selected cytotoxic or transcriptional features (103), this overlap is descriptive and does not establish shared antigen specificity, equivalent pathogenesis, or improved immunotherapy outcomes in DTC. PD-1/PD-L1 is therefore discussed as a regulatory axis relevant to both settings, rather than as evidence that ICI-induced thyroiditis validates an antitumor role for spontaneous HT.

**Table 3:** Comparison of spontaneous Hashimoto’s thyroiditis, immune checkpoint inhibitor-induced thyroiditis, and HT-associated differentiated thyroid cancer according to molecular mechanisms, laboratory abnormalities, and clinical manifestations.

Comparison domain	Spontaneous HT	ICI-induced thyroiditis	HT-associated DTC
Molecular mechanisms	Chronic loss of thyroid-specific tolerance with Th1-skewed T- and B-cell infiltration and autoantibody production, leading to progressive thyrocyte injury (6, 17–19, 47, 48).	ICI-mediated checkpoint disinhibition activates cytotoxic thyroid-directed T cells, including CD8+, cytotoxic memory CD4+, and IL-21-driven CXCR6+CD8+ populations; the latter is not typical of spontaneous HT (46, 93–96).	Autoimmune surveillance and tumor-adapted suppression coexist. Cytotoxic T cells, B cells/TLSs, and antigen presentation may restrain tumors, whereas checkpoint and suppressive immune programs permit escape; direct evidence is mainly PTC-based (61, 74, 80, 86, 103, 105).
Clinical laboratory abnormalities	TPOAb and/or TgAb elevation; thyroid function may be normal initially, progressing to increased TSH and reduced FT4 in overt hypothyroidism (6, 17–19, 47).	Transient thyrotoxicosis (low TSH, high FT4/FT3) may progress to persistent hypothyroidism. Autoantibody positivity is variable (81, 94, 101, 102).	TPOAb/TgAb may remain elevated. TgAb can interfere with postoperative thyroglobulin monitoring and may provide risk information in selected cohorts; TSH requires contextual interpretation (25, 38, 180, 187, 191, 192).
Clinical symptoms and manifestations	Often asymptomatic; possible painless goiter or hypothyroid symptoms, including fatigue, cold intolerance, and weight gain (6, 47).	Often detected during routine monitoring; transient thyrotoxic symptoms may be followed by persistent hypothyroid symptoms after ICI exposure (81, 94, 102).	No specific combined symptom pattern. Many cases are incidentally detected as nodules; symptoms usually reflect HT-related dysfunction or tumor burden (7, 21, 29, 38).

DTC, differentiated thyroid cancer; FT3, free triiodothyronine; FT4, free thyroxine; HT, Hashimoto’s thyroiditis; ICI, immune checkpoint inhibitor; MHC-II, major histocompatibility complex class II; PD-1, programmed cell death protein 1; PTC, papillary thyroid carcinoma; TgAb, anti-thyroglobulin antibody; TLS, tertiary lymphoid structure; TPOAb, anti-thyroid peroxidase antibody; TSH, thyroid-stimulating hormone.

Most clinical evidence regarding ICI-induced thyroiditis derives from patients receiving ICIs for non-thyroid malignancies, whereas most direct clinical and mechanistic evidence regarding HT-associated DTC derives from PTC. The three conditions are therefore compared descriptively and should not be interpreted as mechanistically equivalent.

### Shared antigens and beneficial autoimmunity

The concept of “beneficial autoimmunity” proposes that immune responses against antigens shared by cancer cells and normal tissues may reflect effective anti-tumor immunity, even when they cause collateral tissue damage (104). In HT-associated PTC, this concept helps explain an apparent paradox: chronic autoimmune inflammation may increase tumor detection or early tumor-promoting pressure, whereas established autoimmune responses may also restrain tumor progression. A pivotal animal study showed that the timing of thyroiditis is critical: only pre-existing thyroiditis conferred protection against BRAF V600E-driven PTC, an effect linked to robust intratumoral infiltration by effector memory CD8^+^ T cells and CD19^+^ B cells (105). In this model, mice with established thyroiditis before cancer induction showed longer survival, lower tumor incidence, and less aggressive tumor features (105). In HT-associated PTC tissues, increased IL-10 and MHC-I expression has been observed, while IL-10 treatment increased MHC-I and reduced PD-L1 expression in PTC cell lines and altered T-cell responses in coculture (106). Whether this pathway contributes to favorable clinical outcomes remains unproven.

However, inflammation remains a double-edged process. Transcriptomic clustering suggests that high oxidative stress in thyroid cancer correlates with BRAF mutations and may promote an immunosuppressive microenvironment by excluding CD8^+^ T cells, potentially through Wnt signaling and regulators such as IL1RN (107). Dedifferentiation further reshapes tumor immunogenicity by altering thyroid function-related genes, including thyroglobulin (TG), sodium-iodide symporter (NIS), and thyroid peroxidase (TPO). Genetic and epigenetic changes—including TP53 and TERT promoter alterations, cell-cycle dysregulation, chromatin remodeling, histone modification, and DNA mismatch repair—may generate tumor-associated antigens (TAAs) and tumor-specific antigens (TSAs) (108). In aggressive RAS-driven tumors, tumor-specific alternative splicing may also create neoantigens with potential relevance for cancer vaccines or T-cell therapies (109).

Shared antigenicity may provide a mechanistic bridge between thyroid autoimmunity and anti-tumor immunity. Some tumor epitopes share sequence homology with thyroid antigens; for example, NY-ESO-1 shares homologous epitopes with both TG and thyroid-stimulating hormone receptor (TSHR) (94). p53-related immune regulation further illustrates this duality. Targeted modulation of p53-associated immune pathways may promote anti-tumor immunity while reducing autoimmune toxicity, partly by decreasing activated CD4^+^ PD-1^+^ cells (110). Conversely, wild-type p53 expression in thyroid carcinoma cells can induce MHC class II expression and activate MHC-II-restricted CD4^+^ cytotoxic T-cell responses (111). Recent *in vivo* evidence further indicates that MAPK inhibitor efficacy in BRAF-mutant ATC depends on this MHC-II-restricted CD4^+^ T-cell cytotoxicity (112), and low-dose lenvatinib can promote tumor control through the enrichment of Granzyme B^+^ CD4^+^ T cells in a BRAF/p53 mouse model (113). Histological analyses also suggest that lenvatinib may unmask local cytotoxic immunity, reduce microvessel density, and induce Hashimoto-like inflammatory remodeling in non-cancerous thyroid tissue, creating an acquired autoimmune-like anti-tumor state (114).

Nevertheless, immune escape can emerge when tumors lose immunogenicity or remodel antigen-presentation pathways. Epigenetic silencing may reduce CD4^+^ T-cell surveillance, while distinct immune clusters in PTC may differ in sensitivity to immune checkpoint blockade (ICB). Tumor mutational burden (TMB) is not a straightforward biomarker in PTC: high TMB has been associated with worse prognosis in one study (115), whereas other immune-related signatures, including Immunophenoscore (IPS) and prognostic immune-related signatures (IRS), may better stratify outcomes and radioiodine (RAI) resistance (116–118). Concurrent autoimmunity may also complicate metabolic radiotherapy, as autoimmune thyroiditis can impair RAI uptake and alter TNF-α signaling responses compared with non-autoimmune cases (119). Further evidence from single-cell RNA sequencing suggests that ATC may dedifferentiate from a subset of PTC through CREB3L1-mediated EMT programs, with important implications for relapse risk and immune remodeling (120). Although dedifferentiation may increase tumor immunogenicity in some contexts (121), clinical benefit appears to depend on the immune microenvironment and treatment combinations; for instance, lenvatinib plus pembrolizumab has shown promising responses in aggressive ATC and PDTC, particularly in tumors with high TMB or PD-L1 expression (122). Spatial transcriptomics and mass spectrometry imaging further reveal marked heterogeneity in epitope-specific immunity, ligand-receptor interactions, and chromatin states across PTC foci and metastatic progression (87, 124–129). Together, direct evidence from HT-associated PTC and indirect evidence from advanced thyroid malignancies suggest that immune escape during thyroid cancer progression is not simply the absence of inflammation, but an active remodeling process involving antigenicity, checkpoint signaling, dedifferentiation, spatial heterogeneity, and treatment pressure.

## Spontaneous HT and ICI-induced thyroiditis: distinct immune states and translational lessons

### Translational lessons for biomarkers and redifferentiation therapy

While inflammation is a recognized component of malignancy, recognition of tumor antigens, including those shared with non-malignant thyroid cells, may also drive beneficial autoimmunity (104). Recent transcriptomic and multi-omics studies have refined this concept in PTC. Yan et al. (130) reported that concurrent HT can confound preoperative risk assessment, causing traditional factors such as male sex to lose predictive value for lymph node metastasis (LNM), with metastatic variability in men mainly linked to immune pathway enrichment. Multi-omics profiling further highlighted innate immunity, showing that elevated tumor-infiltrating neutrophils independently predict lateral LNM and may create a pro-metastatic inflammatory niche (131). These findings suggest that conventional clinicopathological variables are insufficient in HT-associated PTC and should be complemented by immune biomarkers.

PD-L1 is a key candidate biomarker. A systematic review confirmed that PD-L1 expression predicts reduced disease-free survival in PTC and is associated with both HT and BRAF V600E mutations, although it does not consistently correlate with overall survival (132). Other studies comparing PTC microenvironments found that HT is associated with greater overall T-cell infiltration, whereas tumors without HT may display a more focused and homogeneous anti-tumor T-cell repertoire across regions (61, 74). To test the antigenic mimicry hypothesis and guide personalized T-cell receptor (TCR)-based strategies, single-cell TCR sequencing can now track whether thyroid-destructive clones in HT overlap with tumor-reactive clones (133). Patient-derived organoids (PDOs) provide a complementary platform to study these anti-tumor mechanisms, as they preserve tumor genomic features and native immunogenicity (134). These observations challenge the assumption that BRAF-mutant PTC is uniformly immunologically cold; in selected contexts, it displays an immune-hot phenotype that supports exploration of immune checkpoint-based strategies (135). However, increased tumor mutational burden (TMB) can paradoxically coexist with an immunosuppressive microenvironment marked by reduced cytotoxic CD8^+^ T cells and increased resting NK cells and DCs (71). Furthermore, LNM correlates with increased M2 macrophages, mast cells, and Tregs, alongside decreased plasma cells, irrespective of HT status (136).

Direct evidence linking concurrent HT to therapeutic response remains limited and is derived predominantly from PTC-based clinical or experimental studies. The following discussion therefore distinguishes direct PTC-based evidence from thyroid-lineage contextual evidence obtained from PDTC, ATC, and other advanced thyroid malignancies that were generally not stratified according to HT status. These advanced-tumor studies are included to illustrate how high-grade progression and dedifferentiation may alter immune-cell organization, antigen presentation, checkpoint dependence, treatment resistance, and therapeutic target expression. They should not be interpreted as demonstrating treatment efficacy specifically in HT-associated PTC or DTC. Immune phenotyping across thyroid cancer subtypes nevertheless underscores the importance of differentiation state. Thyroid cancers have been broadly classified into an ATC-like immune-hot phenotype, enriched for immune infiltration and inhibitory checkpoints, and a PDTC-like immune-cold phenotype marked by immune exclusion (137, 138). ATC may also exhibit distinct stromal and immune microenvironmental states (139), and a subset of ATCs may arise from PTC through stepwise genomic, transcriptomic, and immune-microenvironmental reprogramming (140, 141). In selected ATCs, CXCL13^+^ T cells and early tertiary lymphoid structure-like features may be associated with greater sensitivity to immunotherapy (121).

Dedifferentiation strongly shapes immune escape and treatment resistance. Across advanced thyroid cancers, enrichment of regulatory T cells and exhausted T-cell states may contribute to resistance to anti-PD-1 therapy (90, 142–144). A low thyroid differentiation score correlates with increased ImmuneScore and immunosuppressive markers, particularly in BRAF V600E-positive tumors (145, 146). MAPK-driven dedifferentiation also contributes to radioiodine refractoriness through reduced sodium–iodide symporter and TSHR expression (147). In parallel, BRAF V600E can promote immune suppression through TBX3-dependent recruitment of myeloid-derived suppressor cells (148). These findings link loss of thyroid differentiation to both radioiodine resistance and impaired immune control.

Therapeutically, these observations support biomarker-stratified combination approaches. A systematic review of 33 trials suggests that immune checkpoint inhibitors have potential activity in thyroid cancer, although the absence of phase III evidence underscores the need for careful molecular and immune selection (149, 150). Preclinical studies further indicate that targeted therapy may exert context-dependent effects on antitumor immunity, either enhancing immune activation or promoting compensatory immunosuppressive remodeling (152, 153). Restoration of antigen presentation may therefore represent an important complementary strategy. MHC-I expression may influence the immunostimulatory effects of radiopharmaceutical therapy (154), and MHC-I loss in PTC can be reversed by selumetinib or interferon to enhance T-cell activation (155). In BRAF-mutant ATC, MHC-II/CIITA-dependent CD4^+^ T-cell responses also influence sensitivity to MAPK inhibition (112). These observations provide a rationale for combining redifferentiation, targeted therapy, and immunotherapy, but their relevance to HT-associated DTC remains indirect and hypothesis-generating.

### Immune checkpoint and adoptive cell therapies

Preclinical evidence suggests that the efficacy of immune checkpoint inhibitors (ICIs) in PTC heavily depends on the pre-existing immune context. In a murine model examining the timing of iodine-exacerbated thyroiditis relative to PTC induction, ICI treatment reduced tumor incidence in the concomitant thyroiditis group, but not in the pre-existing thyroiditis or no-thyroiditis groups (156). These findings indicate a context-dependent treatment response rather than a general benefit of thyroidal immune preconditioning. In BRAF V600E-mutated ATC, combining dabrafenib/trametinib with anti-PD-1 therapy has improved overall and progression-free survival; however, data specifically addressing patients with coexisting HT remain limited (134, 135, 157–159). Even when combination strategies demonstrate activity, adaptive resistance mechanisms, such as the compensatory expansion of FOXP3^+^ regulatory T cells, may attenuate long-term efficacy. These observations support the continued evaluation of combinations that enhance effector T-cell activity while actively suppressing inhibitory cell populations. Consequently, contemporary ICI trials in thyroid cancer have increasingly shifted toward subtype-specific and biomarker-guided designs, incorporating lenvatinib, pembrolizumab, and emerging immunomodulatory agents (158).

Direct evidence for CAR-T-cell, CAR-NK-cell, or other adoptive-cell therapies specifically in HT-associated DTC is currently unavailable; the following approaches are therefore discussed only as emerging therapeutic strategies in advanced thyroid cancer. Adoptive cell therapy (ACT) offers a complementary strategy, particularly because thyroid cancer allows for the selection of tissue-restricted targets following thyroidectomy. Analyses of patient-derived tumor-infiltrating lymphocytes have identified tumor-reactive T-cell receptors (TCRs) specific for neoantigens such as BRAF V600E and KRAS G12D/V (151). Further characterization of TCR specificities in DTC may accelerate the development of TIL- or TCR-engineered therapies. Recent studies have evaluated TSHR-directed and other CAR-T/CAR-NK strategies for advanced thyroid cancer (160–163). Early data support feasibility, but the efficacy of TSHR-targeted approaches depends on preserved thyroid differentiation and target expression; alternative targets such as CSPG4 and B7-H3 may remain relevant after TSHR loss (161, 162, 164).

Demethylating agents may broaden T-cell targetability by inducing NY-ESO-1 expression in aggressive thyroid cancer, although clinical translation remains uncertain (165). ACT in thyroid cancer remains limited by antigen and spatial heterogeneity, poor tumor infiltration, immunosuppressive microenvironments, and thyroid-directed toxicity (151, 166, 167). TSHR-directed CAR-T/NK therapy is mechanistically distinct from ICI-associated thyroiditis because it induces target-specific rather than checkpoint-disinhibited cytotoxicity. To facilitate a clinically oriented comparison, **Table 3** summarizes spontaneous HT, ICI-induced thyroiditis, and HT-associated DTC according to their molecular mechanisms, abnormalities in clinical laboratory parameters, and clinical symptoms or manifestations. Because these conditions differ in their initiating triggers, evidence base, and clinical context, this comparison is intended to clarify their distinctions rather than imply mechanistic equivalence.

## Integrative model of the spear-and-shield balance

### Stage-specific transition from inflammation to immune surveillance

Chronic inflammation can promote carcinogenesis by disrupting tissue homeostasis and increasing reactive oxygen species (ROS)-mediated genomic instability. Persistent activation of NF-κB and STAT3 signaling supports cell proliferation and survival, and may also favor an immunosuppressive tumor microenvironment. In HT, this oxidative and cytokine-rich milieu may contribute to the proposed “spear” through inflammation-associated selective pressure; however, excessive oxidative stress may also exceed the adaptive antioxidant capacity of tumor cells and promote ferroptosis or metabolic dormancy, thereby counterbalancing oncogenic progression in some contexts (168). Evidence from inflammation-associated cancer models and anti-inflammatory interventions supports a pathogenic link between sustained inflammation and tumorigenesis (169–172). In a distinct setting of immune dysregulation, a longitudinal study of adult-onset immunodeficiency driven by anti-IFN-γ autoantibodies reported a 25% malignancy rate, suggesting that chronic infection and persistent autoimmunity can create permissive conditions for neoplastic transformation (173). These observations indicate that inflammation alone is insufficient to explain tumor initiation in all HT patients. Instead, endocrine signals, genetic background, immune composition, and inflammatory polarity may jointly shape whether chronic thyroiditis promotes or restrains tumor development.

One possible explanation for this divergence is the balance between pro-inflammatory cytokine-dominant programs and immunoregulatory or wound-healing programs. In tumor-dormancy models, pro-inflammatory responses may help maintain tumor-cell dormancy in selected contexts, whereas persistent, non-resolving inflammation accompanied by wound-healing signals may facilitate dormant-cell reactivation and recurrence (174). HT is frequently characterized by Th1-skewed inflammation and IFN-γ/TNF-α-driven CXCL10/CXCR3 signaling (6, 19). Although this inflammatory axis contributes to autoimmune thyroiditis, its persistent dysregulation may also promote thyroid cancer growth, invasion, and metastasis (19). Thus, the same inflammatory circuits that initiate thyroid-directed autoimmunity may exert different effects depending on timing, cellular context, and tumor genotype.

Conversely, several studies support a tumor-restraining role for pre-existing thyroiditis. In a murine model, pre-existing thyroiditis was associated with abundant effector memory CD8^+^ T cells and CD19^+^ B cells, immune features linked to favorable prognosis (105, 175). Chronic inflammation may also promote tertiary lymphoid structure (TLS) formation, facilitating local antigen presentation and adaptive immune responses (176). In human PTC, trajectory and RNA-velocity analyses suggested migration potential of B cells from HT-affected adjacent tissue toward tumor tissue, while inferred cell–cell interactions implicated myeloid cells in B-cell recruitment (74). Consistently, HT has been reported to remodel the tumor immune microenvironment from a pro-metastatic immune-excluded state toward an inflamed phenotype characterized by STAT6 upregulation and cytotoxic infiltration (80). Additional mechanisms may include IL-17A-mediated enhancement of MHC-I expression on cancer cells (177) and suppression of epithelial-mesenchymal transition, reflected by increased E-cadherin and TGF-β alongside reduced N-cadherin in tumors with concurrent HT (178). PD-1 blockade models demonstrate that checkpoint disinhibition can induce destructive thyroiditis (95), but they do not establish that spontaneous HT restrains thyroid cancer. The tumor-restraining component of the present model is instead supported by HT-associated PTC-specific evidence, including pre-existing thyroiditis models and human studies of cytotoxic immune infiltration and B-cell migration. Together with evidence that RET/PTC1 can confer a survival advantage within an inflammatory environment (55), these observations support a provisional bidirectional model in which chronic inflammation may select for oncogenic clones, whereas pre-existing thyroid-directed immunity may contribute to tumor containment in selected HT-associated PTC contexts (179).

### TSH–TSHR signaling in the autoimmune thyroid tumor microenvironment

TSH–TSHR signaling represents an important endocrine-immune interface in HT-associated DTC. In TSHR-expressing thyroid cancer cells, TSH can promote proliferation, invasion, and immune evasion, partly by inducing PD-L1 expression through the adenylyl cyclase (AC)-PKA-JNK-c-Jun pathway. Pharmacological inhibition of TSHR signaling can reverse this immunosuppressive effect, suggesting that TSH suppression may have therapeutic relevance when combined with immune checkpoint blockade (180). Although TSH is widely recognized as a growth-promoting factor in DTC, tumor responsiveness to TSH is highly heterogeneous (19, 47). Higher baseline TSH has also been associated with an increased risk of thyroid dysfunction after ICI therapy, further linking the TSH axis to immune-endocrine susceptibility (94).

This heterogeneity may reflect the dependence of TSH signaling on tumor differentiation status and TSHR expression. Abaandou et al. proposed that TSH should be viewed primarily as a tumor promoter rather than a *de novo* initiator, because its oncogenic effect depends on preserved differentiation and sufficient TSHR density, both of which often decline in aggressive or dedifferentiated tumors (181). Genetic and epigenetic alterations may further modify this response (181, 182). A GWAS identified 112 putative causal genes linking TSH regulation with tumor-related pathways, including VEGF-mediated angiogenesis and coagulation (183). *In vivo* evidence also suggests that TSHR signaling may preferentially drive goiter formation rather than thyroid carcinogenesis in the absence of IGF-1 or insulin signaling (181). However, this view is not absolute. Eszlinger et al. showed in a TSHR D633H knock-in mouse model that constitutive TSHR signaling alone can induce PTC through ERK/MAPK activation, mediated in part by downregulation of the inhibitory BRAF p.S766 site (184). Consistently, systematic reviews involving 23,374 PTC cases and 76,301 controls reported a positive association between higher TSH concentrations and thyroid cancer incidence (185, 186).

In autoimmune thyroid disease (AITD), however, the relationship between TSH and malignancy appears less straightforward. A large multicenter analysis showed that the association between high-normal TSH and malignancy risk was no longer observed in patients with AITD (187). This dissociation suggests that the inflammatory and cytotoxic immune microenvironment of HT may counterbalance, or at least obscure, the canonical tumor-promoting effects of TSH. In this setting, TSH levels alone may be an unreliable predictor of malignancy risk. Persistent tumor progression despite TSH suppression may also reflect TSH-independent mechanisms, including the proposed integrin αvβ3 pathway, through which thyroid hormones such as T4 and T3 can activate MAPK-mediated proliferation via membrane receptors and bypass the TSH–TSHR axis (181). Conversely, lower TSH has also been associated with tumor onset in another cohort study, further emphasizing the context-dependent nature of this endocrine signal (188).

Beyond its tumor-cell-intrinsic effects, evidence for direct immune-cell modulation by TSH remains limited in thyroid cancer. Local TSH/TSHR signaling has been shown to promote CD8^+^ T-cell exhaustion through the TSHR-PKA-CREB axis, with upregulation of inhibitory receptors such as PD-1 and TIM-3, in colorectal carcinoma; therefore, this mechanism should be presented as hypothesis-generating rather than as definitive evidence for HT-associated DTC (189). At the biochemical level, PTC without concurrent HT has been reported to exhibit higher oxidative stress, Ki-67 expression, and VEGF levels than PTC with HT, providing a possible functional explanation for the more favorable prognosis often observed in HT-associated tumors (190). Single-cell analysis further identified HT-associated cell populations (HASCs) that may establish a TSH-suppressive microenvironment through the MIF-CD74/CXCR4 signaling axis (61). These findings may help explain why elevated TSH in subclinical hypothyroidism does not consistently translate into tumor initiation among patients with HT.

Clinically, an autoimmune background also complicates biomarker interpretation. A prospective multicenter study showed that patients with thyroid cancer and coexisting HT were more likely to show abnormal biochemical markers suggestive of recurrence one year after treatment, but this did not translate into a higher risk of structural disease recurrence (38). These abnormalities may therefore reflect inflammation-related interference rather than true tumor regrowth. Nevertheless, antibody profiles should not be dismissed. Compared with TPOAb, markedly elevated TgAb levels (>1,150 IU/ml) have been associated with central lymph node metastasis and may provide additional risk information in selected patients (70, 191).

## Discussion and future directions

The central conclusion of this review is that concurrent Hashimoto’s thyroiditis (HT) modifies the immune microenvironment of differentiated thyroid cancer (DTC), but does not exert a uniformly protective or tumor-promoting effect. Because most direct clinical and mechanistic evidence is derived from papillary thyroid carcinoma (PTC), the conclusions of this review should be interpreted primarily in the context of HT-associated PTC; evidence for HT-associated follicular and oncocytic thyroid carcinomas remains limited. Clinically, concurrent HT is frequently associated with smaller tumors and relatively favorable recurrence-related features, but these associations vary according to age, tumor genotype, and clinical setting and may be partly influenced by intensified surveillance (7, 17, 21, 23, 29, 33–38). HT should therefore not be considered an independent favorable prognostic marker without accounting for its diagnostic definition, timing, surveillance intensity, and tumor context.

The most directly supported immunological conclusion is that HT-associated PTC commonly exhibits an immune-infiltrated but functionally heterogeneous tumor microenvironment. Increased CD8^+^ T-cell infiltration and STAT6-associated immune remodeling have been observed in HT-associated PTC, while single-cell analyses have suggested migration potential of B cells from inflamed thyroid tissue toward the tumor (74, 80). In a preclinical model, thyroiditis established before BRAF V600E-driven PTC induction was associated with increased effector-memory CD8^+^ T cells and CD19^+^ B cells, reduced tumor incidence, and improved survival (105). These findings support the possibility that pre-existing thyroid-directed immunity contributes to tumor containment in selected settings. However, immune-cell abundance alone does not establish effective tumor surveillance. Regulatory T cells, suppressive myeloid populations, dysfunctional dendritic-cell states, and checkpoint activation may coexist within the same inflamed microenvironment (83, 84, 86). The effect of HT is therefore more likely to depend on immune-cell phenotype, antigen specificity, spatial organization, B-cell/TLS organization, and preservation of antigen presentation than on lymphocyte density alone.

Several components of the proposed spear-and-shield framework remain biologically plausible but incompletely validated. Chronic inflammation and oxidative stress may create selective pressure favoring oncogenic clones, and RET/PTC-related signaling provides a potential mechanism through which transformed thyrocytes may adapt to an inflammatory environment (55–57). Nevertheless, direct longitudinal evidence demonstrating that spontaneous HT drives clonal evolution in humans is lacking. Similarly, shared-antigen cross-reactivity may connect thyroid autoimmunity with tumor recognition, but shared thyroid- and tumor-reactive T- or B-cell clones have not yet been directly demonstrated in paired human tissues. TSH–TSHR signaling can induce tumor-cell PD-L1 expression in thyroid cancer (180), whereas direct TSH-mediated CD8^+^ T-cell exhaustion has been demonstrated mainly in a non-thyroid malignancy model (189) and remains hypothesis-generating in DTC.

ICI-induced thyroiditis should be viewed only as a comparative human model of treatment-induced checkpoint disinhibition and thyroid-directed immune toxicity. Because most clinical evidence derives from patients with non-thyroid malignancies, it does not establish that spontaneous HT enhances tumor immune surveillance or predicts immunotherapy benefit in DTC (46, 81, 93–96). Likewise, therapeutic findings from poorly differentiated thyroid carcinoma, anaplastic thyroid carcinoma, and adoptive-cell-therapy studies, most of which were not stratified according to HT status, should be regarded as indirect and hypothesis-generating rather than as evidence of clinical efficacy in HT-associated DTC (121, 122, 137, 160–164). Accordingly, the spear-and-shield framework should be regarded as a conceptual synthesis of current evidence rather than as an established causal sequence.

Future studies should prioritize prospective and spatially resolved evaluation of HT-associated immunity. HT should be defined according to timing, histology, antibody profile, thyroid functional status, and ultrasound findings, together with documentation of iodine exposure, surveillance intensity, tumor genotype, histological subtype, differentiation state, and treatment course. Matched thyroiditis tissue, primary tumors, lymph-node metastases, and distant lesions should be analyzed using single-cell and spatial transcriptomics, TCR/BCR repertoire sequencing, HLA typing, immunopeptidomics, and multiplex imaging (125, 133, 167, 193–196). These approaches should determine whether immune clones are shared between inflamed thyroid tissue and tumor, whether HT-associated immune states persist or are remodeled during progression and dedifferentiation, and whether they predict treatment response.

The conclusions of this review should also be interpreted in light of its narrative design. Although a structured literature search and explicit eligibility criteria were used, no formal risk-of-bias assessment or certainty-of-evidence grading was performed. Until more direct prospective and mechanistic evidence becomes available, HT is best regarded as a context-dependent immune modifier rather than a uniform risk or prognostic factor. **Figure 2** summarizes this context-dependent model and its proposed translational implications, which remain to be prospectively validated.

**Figure 2:**
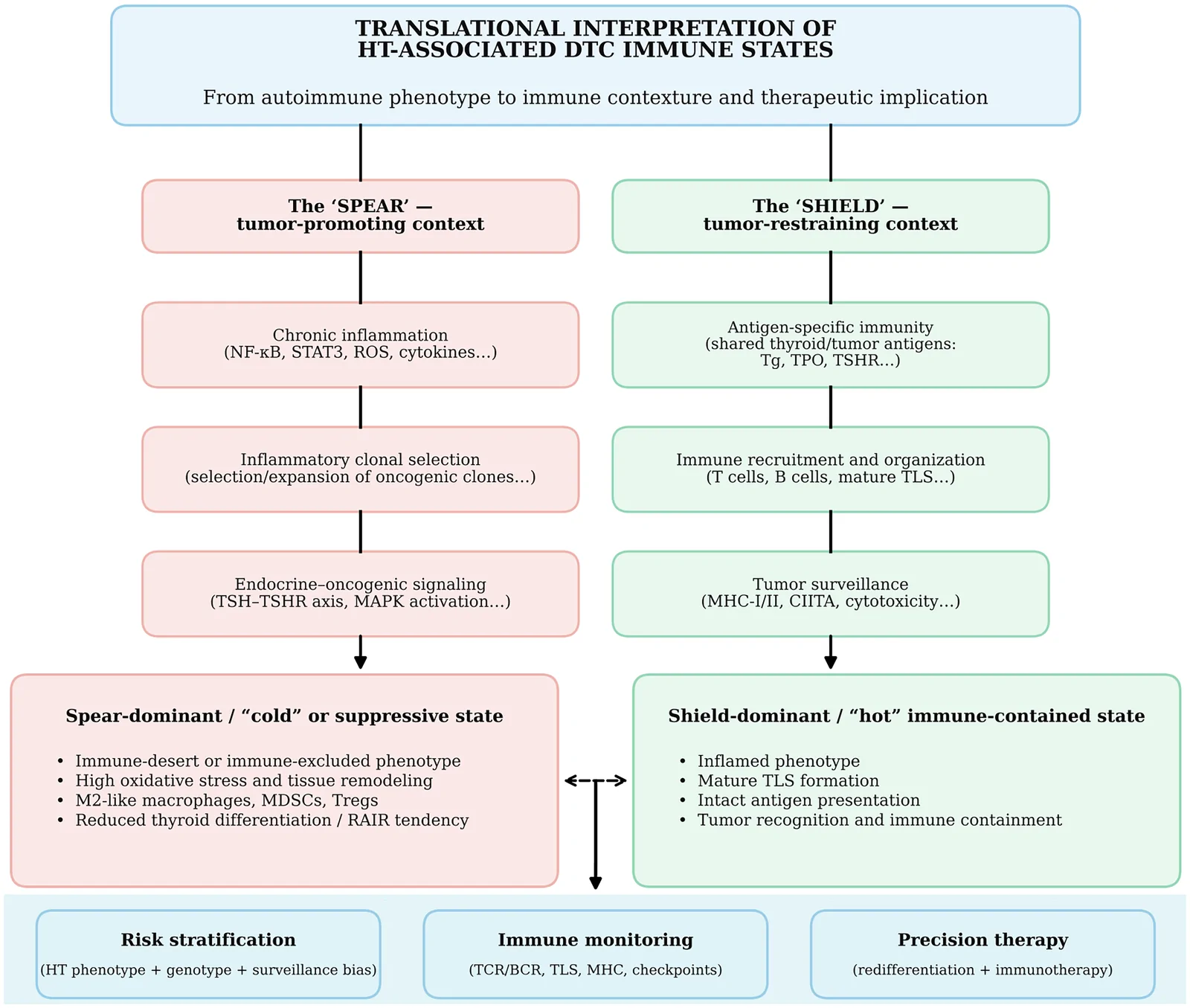
Translational interpretation of HT-associated DTC immune states. This simplified workflow translates the spear-and-shield model into clinically and experimentally tractable immune states. A spear-dominant context is characterized by chronic inflammation, inflammatory clonal selection, endocrine–oncogenic signaling, immune exclusion, myeloid/Treg-mediated suppression, reduced thyroid differentiation, and radioiodine-refractory tendency. A shield-dominant context is characterized by antigen-specific immunity, organized T-cell and B-cell recruitment, mature TLS formation, intact MHC-I/MHC-II/CIITA antigen presentation, and cytotoxic effector function. Integration of HT phenotype, tumor genotype, surveillance intensity, immune spatial organization, differentiation state, and checkpoint activation may support risk stratification, immune monitoring, and rational combinations of redifferentiation therapy with immunotherapy.
