# Supplementary material for: Hashimoto’s thyroiditis in differentiated thyroid cancer: autoimmune inflammation, immune surveillance, and tumor escape
Source: Front Immunol. 2026 Oct 16;17:1945791. doi: 10.3389/fimmu.2026.1945791 (PMC13720714; doi:10.3389/fimmu.2026.1945791)
Supplement: Supplementary file 1 [file Table1.docx]

**Supporting Information of**

**Hashimoto’s Thyroiditis in Differentiated Thyroid Cancer: Autoimmune Inflammation, Immune Surveillance, and Tumor Escape**

Zijian Zhang^1†^, Likun Cui^2†^, Quan Dai^2^, Chuanshi He^2^, Li Han^2^, Min Zhuang^1^, Lu Wang^2^, Ziyue Hu^2^, Xiaoxiao Xie^2^, Yuping Shen^2^, Jinshun Xu^2*^, Man Lu^2*^

^1^ Department of Ultrasound, Sichuan Clinical Research Center for Cancer, Sichuan Cancer Hospital & Institute, Sichuan Cancer Center, School of Medicine, University of Electronic Science and Technology of China, Chengdu, China.

^2^ Department of Ultrasound, Sichuan Clinical Research Center for Cancer, Sichuan Cancer Hospital & Institute, Sichuan Cancer Center, University of Electronic Science and Technology of China, Chengdu, China.

^†^These authors have contributed equally to this work and share first authorship.

*Corresponding author

Email: luman@uestc.edu.cn (Man Lu); xujinshun@uestc.edu.cn (Jinshun Xu)

**Table S1. Database-specific literature-search strategy for the critical narrative review**

**Table S1A. Search concept blocks**

| **Search block** | **Concept** | **Exact free-text terms** |
| --- | --- | --- |
| **A** | Hashimoto’s thyroiditis and thyroid autoimmunity | “Hashimoto thyroiditis” OR “Hashimoto’s thyroiditis” OR “Hashimoto disease” OR “chronic lymphocytic thyroiditis” OR “autoimmune thyroiditis” OR “thyroid autoimmunity” OR “autoimmune thyroid disease” |
| **B** | Follicular-cell-derived thyroid cancer | “thyroid neoplasm*” OR “thyroid cancer*” OR “thyroid carcinoma*” OR “differentiated thyroid cancer” OR “differentiated thyroid carcinoma” OR “papillary thyroid carcinoma” OR “papillary thyroid cancer” OR “follicular thyroid carcinoma” OR “follicular thyroid cancer” OR “oncocytic thyroid carcinoma” OR “Hürthle cell carcinoma” OR “Hurthle cell carcinoma” |
| **C** | Epidemiology, surveillance, and clinical outcomes | incidence OR prevalence OR coexist* OR associat* OR risk OR surveillance OR overdiagnos* OR prognos* OR recurren* OR metastas* OR survival OR clinicopatholog* |
| **D** | Tumor genomics and inflammatory clonal selection | BRAF OR “BRAF V600E” OR BRAFV600E OR RAS OR “RET/PTC” OR “RET fusion*” OR NTRK OR ALK OR ROS1 OR TERT OR “gene fusion*” OR “genomic alteration*” OR “clonal selection” OR MAPK OR “PI3K-AKT” |
| **E** | Tumor immune microenvironment and immune-cell populations | “tumor immune microenvironment” OR “tumour immune microenvironment” OR “immune surveillance” OR “immune escape” OR immun* OR inflamm* OR “T cell*” OR CD8 OR CD4 OR “B cell*” OR macrophage* OR “dendritic cell*” OR “natural killer” OR Treg* OR MDSC* OR cytokine* OR chemokine* |
| **F** | Antigen presentation, clonality, and spatial immune organization | “antigen presentation” OR “MHC class I” OR “MHC-I” OR “MHC class II” OR “MHC-II” OR CIITA OR “T-cell receptor” OR TCR OR “B-cell receptor” OR BCR OR clonality OR “tertiary lymphoid structure*” OR TLS OR “single-cell” OR “single cell” OR “spatial transcriptomic*” OR “spatial profil*” OR immunopeptidom* |
| **G** | Immune checkpoints and ICI-induced thyroiditis | “PD-1” OR “PD-L1” OR checkpoint* OR “immune checkpoint inhibitor*” OR “checkpoint inhibitor-induced thyroiditis” OR “immune checkpoint inhibitor-induced thyroiditis” OR “ICI-induced thyroiditis” OR “immune-related adverse event*” OR irAE* |
| **H** | TSH–TSHR endocrine–immune signaling | TSH OR thyrotropin OR TSHR OR “thyroid-stimulating hormone receptor” OR “thyroid stimulating hormone receptor” |
| **I** | Differentiation, radioiodine refractoriness, and therapy | differentiat* OR dedifferentiat* OR “thyroid differentiation score” OR NIS OR “sodium iodide symporter” OR “radioiodine refractory” OR “radioiodine-refractory” OR redifferentiat* OR immunotherap* OR “CAR-T” OR “CAR NK” OR “chimeric antigen receptor” OR vaccine* OR lenvatinib OR pembrolizumab OR selumetinib OR “MAPK inhibitor*” |

**Table S1B. Search combinations**

| **Search number** | **Search objective** | **Combination** |
| --- | --- | --- |
| Search 1 | Broad identification of studies directly addressing HT and thyroid cancer | A AND B |
| Search 2 | Epidemiological, clinicopathological, and prognostic evidence | A AND B AND C |
| Search 3 | Genomic drivers and inflammation-related clonal selection | A AND B AND D |
| Search 4 | HT-associated tumor immune microenvironment | A AND B AND (E OR F) |
| Search 5 | Spontaneous HT and ICI-induced thyroiditis as comparative immune states | A AND G |
| Search 6 | TSH–TSHR signaling in thyroid cancer | B AND H |
| Search 7 | Differentiation, radioiodine refractoriness, and therapeutic studies | B AND I |
| Search 8 | General thyroid cancer mechanistic context not restricted to concurrent HT | B AND (D OR E OR F OR G OR H OR I) |

*Search 1 was used as the primary disease-association search. Searches 2–7 were used to retrieve domain-specific evidence. Search 8 was used selectively to identify mechanistic studies in thyroid cancer that were not explicitly indexed using HT-related terms. Studies involving poorly differentiated or anaplastic thyroid carcinoma, or non-thyroid malignancies, were retained only when they directly informed mechanisms relevant to the conceptual framework and were interpreted as indirect or hypothesis-generating evidence.*

**Table S1C. Databases and search records**

| **Database** | **Coverage** | **Final search date** | **Search strategy** |
| --- | --- | --- | --- |
| **PubMed/MEDLINE** | From database inception to 22 July 2026 | 22 July 2026 | Searches 1–8 |
| **Web of Science Core Collection** | From database inception to 22 July 2026 | 22 July 2026 | Searches 1–8 |
| **Scopus** | From database inception to 22 July 2026 | 22 July 2026 | Searches 1–8 |
| **Backward and forward citation tracking** | Not applicable | 22 July 2026 | Reference-list and citation screening |

*The review was designed as a critical narrative synthesis rather than a systematic review; therefore, PRISMA-style quantitative record accounting was not undertaken. Database-specific search terms and combinations are reported in Supplementary Tables S1A and S1B.*
